# Radiation-induced sarcomas: A single referral cancer center experience and literature review

**DOI:** 10.3389/fonc.2022.986123

**Published:** 2022-09-30

**Authors:** Simona Laurino, Ludmila Carmen Omer, Francesco Albano, Graziella Marino, Antonella Bianculli, Angela Pia Solazzo, Alessandro Sgambato, Geppino Falco, Sabino Russi, Anna Maria Bochicchio

**Affiliations:** ^1^ Laboratory of Preclinical and Translational Research, Istituto di Ricovero e Cura a Carattere Scientifico (IRCCS) CROB Centro di Riferimento Oncologico della Basilicata, Rionero in Vulture, Italy; ^2^ Trial Office, Istituto di Ricovero e Cura a Carattere Scientifico (IRCCS) CROB Centro di Riferimento Oncologico della Basilicata, Rionero in Vulture, Italy; ^3^ Department of Breast Surgery, Istituto di Ricovero e Cura a Carattere Scientifico (IRCCS) CROB Centro di Riferimento Oncologico della Basilicata, Rionero in Vulture, Italy; ^4^ Radiotherapy Unit, Istituto di Ricovero e Cura a Carattere Scientifico (IRCCS) CROB Centro di Riferimento Oncologico della Basilicata, Rionero in Vulture, Italy; ^5^ Department of Biology, University of Naples Federico II, Naples, Italy; ^6^ Multispecialty Tumor Board, Istituto di Ricovero e Cura a Carattere Scientifico (IRCCS) CROB Centro di Riferimento Oncologico della Basilicata, Rionero in Vulture, Italy

**Keywords:** breast cancer, head and neck cancer, radiation-induced sarcoma, radiotherapy, long-term radiation effects

## Abstract

**Background and objective:**

The oncogenic effect of ionizing radiation is widely known. Sarcomas developing after radiation therapy (RT), termed “iatrogenic disease of success”, represent a growing problem, since the advancements in cancer management and screening programs have increased the number of long-term cancer survivors. Although many patients have been treated with radiation therapy, only few data are available on radiation-induced sarcomas (RIS).

**Methods:**

We examined the medical and radiological records of 186 patients with histologically proven soft tissue and bone sarcomas, which referred to IRCCS CROB Centro di Riferimento Oncologico della Basilicata from January 2009 to May 2022. Among them, seven patients received a histological diagnosis of secondary RIS, according to Cahan’s criteria. Clinicopathological features and treatment follow-up data of RIS patients were retrospectively analyzed.

**Results:**

Among these secondary RIS, five arose in irradiated breast cancer (5/2,570, 0.19%) and two in irradiated head and neck cancer (2/1,986, 0.10%) patients, with a mean onset latency time of 7.3 years.* *The histology of RIS was one desmoid tumor, two angiosarcomas, one chondrosarcoma, two leiomyosarcomas, and one undifferentiated pleomorphic sarcoma. Out of the seven RIS, one received radiotherapy, one received electrochemotherapy (ECT), one received a second-line chemotherapy, three were subjected to three lines of chemotherapy, and one underwent radiofrequency ablation, chemotherapy, and ECT. Median survival time is 36 months. No significant survival differences were found stratifying patients for age at RT, latency time, and age at RIS diagnosis.

**Conclusions:**

RIS represents a possible complication for long-survivor cancer patients. Therefore, adherence to a strict follow-up after the radiation treatment is recommended to allow early diagnosis and optimal management of RIS patients. After the planned follow-up period, considering the long-term risk to develop a RIS, a specific multispecialty survivorship care plan could be of benefit for patients.

## Introduction

Radiation therapy (RT) represents the main treatment strategy for more than half of cancer patients (1–3), since it entails improvement of the survival rates and long-term overall survival in many types of cancer. Therefore, the employment of this treatment option is growing. Indeed, as an example, a Korean study reported a 65% increase in cancer patients who underwent RT from 2006 to 2013 (4). Despite these undoubted benefits, RT is found to be associated with the onset of a rare iatrogenic malignancy, known as “radiation-induced sarcoma” (RIS), which represents about 3% of all soft tissue sarcomas (5). This adverse event is characterized by poor 5-year overall survival, ranging from 10% to 36% in relation to disease stage at diagnosis (1). Therefore, RIS is considered an arduous challenge for physicians. It also represents a growing clinical problem, likely associated with the increasing number of long-term cancer survivors determined by the advancements in cancer screening programs and patient management (6, 7).

The first cases of sarcoma following RT were observed in 1922 by Beck and Marsch in patients irradiated to treat tuberculous bone disease (8, 9). Subsequently, in 1936, Warren and Sommer described complications after irradiation of breast carcinoma in 81 patients (9). In 1948, based on 11 cases of post-radiation osteosarcoma (PRS), Cahan and Woodard defined the following criteria for RIS diagnosis (10):

a) No evidence of the new tumor at RT time;
b) Sarcoma arises in the irradiated field;
c) Relatively long latency period before sarcoma onset; and
d) Histologically proven sarcoma.

A large analysis of the Surveillance, Epidemiology, and End Results (SEER) registries found a 257% increased risk of secondary bone sarcoma in patients who received radiotherapy compared to the general population (11). Recently, these data were examined by Snow et al., who reported that, after cervical cancer, breast cancer has the highest risk of RIS (88.2% and 78.3%, respectively) (12). RIS after breast cancer RT shows a wide range of histopathologic subtypes, among which malignant fibrous histiocytoma is the most common. Less frequent findings include leiomyosarcoma, liposarcoma, fibrosarcoma, and angiosarcoma, and rarely chondrosarcoma and osteosarcoma. These secondary RIS are usually high-grade tumors variable in size, whose histological features include presence of spindle-shaped tumor cells, hemorrhagic tumor nodules, abundant mitotic figures, and necrosis (13).

RIS of the head and neck also represents a relevant problem since, although rare, they are a lethal consequence of RT. Its average frequency was about 0.15% with a mean latency period, the interval between RT on the primary lesion and the onset of secondary sarcoma, of about 11 years. Histologically, RIS of the head and neck are mainly ascribable to osteosarcoma and fibrosarcoma (14).

Here, we performed a retrospective study on patients’ records to investigate the clinical and pathological features of RIS cases that accessed IRCCS CROB Centro di Riferimento Oncologico della Basilicata from 2009 to 2022.

## Materials and methods

### Patient cohort and data collection

We examined the medical record of all histologically diagnosed sarcoma in patients managed from 2009 to 2022, included in both the Basilicata Cancer Registry and the Institutional Electronic Health Dossier. The latter also comprises patients from nearby regions. All data were retrieved from patients who gave their informed consent at the first access or afterwards on request.

Overall, there were 186 cases (85 male and 101 female patients) of sarcomas with a mean age of 59.7 years (range: 15–91 years). At the time of writing (June 2022), patients are followed up in an outpatient setting. The mean time of follow-up is 58.5 months (range: 0.6–380.7 months). Their geographical origin is mainly Basilicata (121), Campania (38), and Puglia (16) (**Table 1**).

**Table 1:** General characteristics and management information of the enrolled sarcoma patients.

Sarcoma patients (from 2009 to 2022)	*N* = 186
**On follow-up**	52
**Sex (M/F)**	85/101
**Age, mean (range)**	59.7 (15–91)
**Patient territorial distribution**	
• **Basilicata** • **Puglia** • **Campania** • **Other**	121 16 38 11
**Center of diagnosis/surgery**	
• **IRCCS CROB**	116
• **Other**	70
**Metastasis at diagnosis (Yes/No)**	74/112
**Disease progression**	73
**Surgical excision**	72
**Chemotherapy**	90
• **Neoadjuvant**	7
• **1st line**	83 (33 patients only one line)
• **2nd line**	50 (I+II)
• **3rd line**	29 (I+II+III)
• **4th line**	15 (I+II+III+IV)
• **5th line**	9 (I+II+II+IV+V)
• **6th line**	3 (I+II+II+IV+V+VI)
**Eribulina**	2
**Olaratumab**	3
**Crizotinib**	1
**Imatinib (Cordoma)**	1
**FOLFIRI/FUFA (carcinosarcoma-MMMT)**	1
**Pomalidomide (Kaposi sarcoma)**	1
**Protocol ISG/SSG (Ewing sarcoma)**	1
**Proton therapy**	1
**Autologous transplant**	1
**Brachytherapy**	2
**Electrochemotherapy**	13
**Radiotherapy**	40
**Palliative**	6
**Adjuvant**	34
**Months of follow-up, mean (range)**	58.5 (0.6–380.7)

### Diagnosis and treatments

The first diagnosis was made at CROB for 116 patients. Seventy-two patients underwent radical surgical excision. Metastases were detected in 74 patients through total body computed tomography (CT) examination at first diagnosis, whereas in 73 cases, new metastatic lesions appeared during follow-up. All patients were treated at our center, except one osteosarcoma patient who was managed at Rizzoli Orthopedic Hospital in Bologna. Several treatment regimens were administered as summarized in **Table 1**. Ninety patients received chemotherapy, 33 of whom received only the first-line setting, 50 patients also received a second-line treatment schedule, and 29 patients received three chemotherapy lines. Notably, off-label and/or targeted therapy regimens were tried. One patient diagnosed with carcinosarcoma (MMMT) received FOLFIRI regimen. In three cases, olaratumab was the first-line treatment. One patient with a myofibroblastic inflammatory tumor of sclera-conjunctiva, positive for anaplastic lymphoma kinase mutation (ALK+), was treated with crizotinib. One patient received imatinib to treat cordoma.

Among the 186 patients, 40 patients received external radiotherapy, 2 cases received brachytherapy, and for 13 patients, electrochemotherapy was employed as local therapy.

### Selection criteria of radiation-induced sarcoma

Criteria by Cahan et al. were used to identify RIS patients (10). For further evaluations, detailed epidemiological, clinical, pathological, and treatment history and survival information were collected.

### Statistical analysis

The association of patients’ overall survival with age at RT, at RIS, or latency time was estimated by log-rank test, after categorization of time in classes and using the *survminer* R package (15). In a similar way, association between RIS risk and age at RT, based on latency time, was explored. Survival curves were then plotted using the Kaplan–Meier method. Hazard ratios were also estimated for each variable by Cox proportional hazards regression model included in the *survival* package (16).

## Results

Among 186 sarcoma patients, we identified seven (3.8%) cases fulfilling Cahan’s criteria. In particular, five RIS arose in the irradiated field of breast cancer patients and two in that of head and neck cancer patients. To better define RIS incidence, we retrospectively analyzed all breast and head and neck primary tumors that underwent radiation therapy. Overall, we found 0.15% (7/4,556) of RIS incidence, in which breast cancer accounts for 0.19% (5/2,570), whereas head and neck cancer accounts for 0.10% (2/1,986). Histological evaluation of RIS found one desmoid tumor, two angiosarcomas, one chondrosarcoma, two leiomyosarcomas, and one undifferentiated pleomorphic sarcoma (**Table 2**). Out of the seven RIS patients, one received radiotherapy, one was treated with electrochemotherapy (ECT), one received a second-line chemotherapy, three underwent three lines of chemotherapy, and one was treated with radiofrequency ablation, chemotherapy, and ECT.

**Table 2:** Histological features, therapeutic management, and follow-up information of radiation-induced sarcomas.

PN	Gender	Primary cancer TNM	Radiotherapy mode/dose	CCRT	Age at RT	Age at RIS	Latency (years)	Location of RIS	Pathology subtypes	Treatment of RIS	Resection Result	Outcome
**1**	M	pT4aN2cM0	IMRT/66 Gy	Y	40	42	2	Nuchal region	Desmoid tumor	CHT	N/A	AWD 08.09.2021 67 months
**2**	F	T2bN3	3D CRT/ 70 Gy	Y	46	53	7	Left sternocleidomastoid muscle	Leiomyosarcoma + pleomorphic areas	S + CHT	R0	DOD 23.08.2016 36 months
**3**	F	pT1N1(10/19) pT1N0	3D CRT photons/ 50 + 10 Gy	Y	63	75	12	Left breast	Chondrosarcoma	S + CHT	R0	DOD 28.09.2018 45 months
**4**	F	pT1cN0	3D CRT photons/ 50 Gy 3D CRT electrons 9 MeV/10 Gy	Y	75	77	2	Left scapulo-humeral	Leiomyosarcoma + undifferentiated high grade pleomorphic sarcoma	RT	R1	DOD 04.01.2014 16 months
**5**	F	pT1cN0	3D CRT photons/ 50 + 10 Gy	Y	61	67	6	Left breast	High grade angiosarcoma	ECT	N/A	DOD 29.10.2019 28 months
**6**	F	pT2N1(8/24)	3D CRT photons/ 50 + 10 Gy	Y	53	67	14	Left armpit + left thoracic wall	High-grade undifferentiated pleomorphic sarcoma (myofibroblastic sarcoma)	CHT	N/A	DOD 15.02.2021 5 months
**7**	F	pT1N1(1/18)	3D CRT photons/ 50 +10 Gy	Y	63	73	10	Left breast	High-grade angiosarcoma	Radiofrequency ablation + CHT + ECT	R0	AWD Last FU 01.03.2022 14 months

TNM stage according to the American Joint Committee of Cancer (AJCC) staging system (7th edition). PN, Patient number; M, Male; F, Female; CCRT, Concurrent Chemoradiotherapy; S + CHT, Surgery + Chemotherapy; RT, Radiotherapy, CHT, Chemotherapy; ECT, Electrochemotherapy; IMRT, Intensity-modulated radiotherapy; 3D CRT, three-dimensional conformal radiation therapy; DOD, Dead of disease; AWD, Alive with disease; N/A, not applicable.

**Figure 1:**
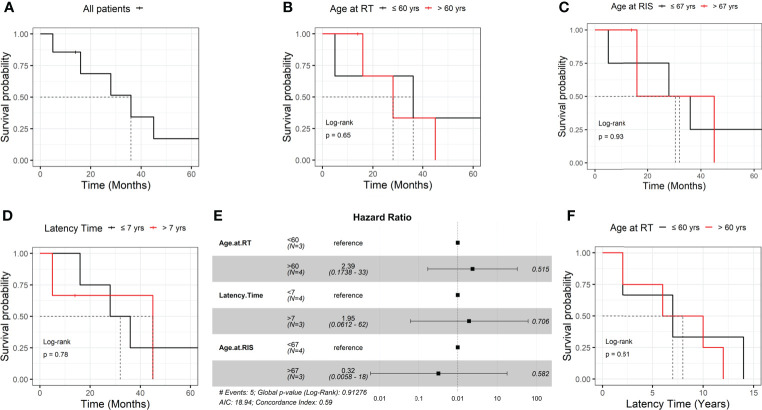
Association of radiation-induced sarcoma patients’ overall survival with different parameters **(A–E)**. Association of radiation-induced sarcoma onset and age at radiation therapy **(F)**. RT, radiation therapy; RIS, radiation-induced sarcoma.

Mean latency time was 7.3 years, ranging from 2 to 14 years. The overall median survival is 36 months (**Figure 1A**). No significant survival differences, likely due to the limited number of RIS cases, were found by stratifying patients for age at RT (36 *vs*. 28 months, ≤60 *vs*. >60 years), latency time (32 *vs*. 45 months, ≤7 *vs*. >7 years), and age at RIS occurrence (32.0 *vs*. 30.5 months, ≤67 *vs*. >67 years) (**Figures 1B–D**). Cox hazard ratio analysis also did not show any association with these variables (**Figure 1E**). Similarly, RIS risk and latency time are not associated with age at RT (7 *vs*. 8 years, ≤60 *vs*. >60 years, respectively) (**Figure 1F**). A detailed case presentation of clinical and pathological findings, including treatments administered and outcomes, is reported below.

### Case 1

A 40-year-old man, in July 2014, had a diagnosis of primary epidermoid carcinoma in the right vocal cord and left lung, stage pT4aN2cM0 and grade G3. In January 2015, both masses were radically excised after neoadjuvant radiotherapy with 66 Gy in 33 fractions on intensity-modulated radiation therapy (IMRT) mode. The patient was free of disease for 15 months until, in March 2016, he received a diagnosis of desmoid tumor in the nuchal area, external to the hot spot of the previously irradiated field. Histological evaluation on a core biopsy described a group of spindle cells included in a collagen matrix arranged as parallel fibers; less than 1/10 HPF (high-power field) mitoses were detected, leading to a diagnosis of extra-abdominal fibromatosis-desmoid tumor. Immunohistochemical staining highlighted cells positive for desmine, SMA (smooth muscle actin), negative for S100, and a Ki67 index of 4%. Angio- and neural invasion was also depicted. After case evaluation by the Institutional Multidisciplinary Tumor Board and its discussion with experts from a rare tumor Comprehensive Cancer Center, the case was considered unsuitable for surgical excision due to the presence of a locally advanced disease infiltrating vascular and nervous structures. The patient was asymptomatic and, considering the poor chemosensitivity of desmoid tumors, he entered on a follow-up care program. In April 2018, due to lesion size increase and localized pain, the patient started a chemotherapy regimen with a combination of two oral drugs, vinorelbine and methotrexate, for 15 weekly cycles. After four months of treatment, due to clinical and radiological disease progression (DP), the patient was treated with second-line chemotherapy consisting of six cycles of a 3-week doxorubicin and dacarbazine regimen. In January 2019, at disease status assessment, the patient had a partial response (PR) and then was addressed to follow-up (every 3 months for the first 2 years, and then every 6 months). At the last follow-up (September 2021), according to RECIST criteria, a further reduction of tumor size was noticed.

### Case 2

The patient is a 46-year-old woman diagnosed in 2006 with nasopharyngeal carcinoma. She was treated with radiotherapy (70 Gy in 35 fractions) combined with weekly cisplatin infusions (five of six cycles regularly administered; the last cycle was suspended due to a suspect of cisplatin‐induced grade 4 pancytopenia). The patient’s clinical history included essential hypertension, hysterectomy for fibromyomas (in 1997), and family history of cancer (a 50-year-old brother with stage III colon cancer). A bilateral hypoacusis as a consequence of radiotherapy was recorded.

After 7 years (September 2013), during routine follow-up, clinical and radiological diagnosis of a mass on the left side of the neck (sternocleidomastoid muscle), referred to as RIS, was made. The patient underwent radical surgery of the left sternocleidomastoid muscle. Histology showed a high-grade mesenchymal neoplasia, consisting of atypical cellular elements, partly fused, with moderate-severe atypia and myogenic differentiation, partly round and oval, sometimes pleomorphic, arranged mostly in bundles and fascicles. Numerous mitotic figures and necrosis areas were observed (Grade 3). Immunohistochemical characterization showed positivity for vimentin, CD34, SMA, and negativity for CD30, CD68, CD31, desmin, and S100. Ki67 index was equal to 70%. Six months later, a local relapse was excised from the left anterior chest wall. After further 6 months, in December 2014, computed tomography (CT) scan showed multiple bilateral lung metastases and the patient received six cycles of first-line chemotherapy based on the combination of gemcitabine and docetaxel. During disease evaluation in April 2015, a strong DP with sternal relapse and pulmonary metastases accompanied by stable lymph nodes was noticed.

Starting from May 2015, six cycles of high-dose ifosfamide continuous infusion were administered as second-line chemotherapy. A minimal partial regression of disease was recorded under CT scan in January 2016. Three months later, an additional CT scan showed lung metastases progression, and dacarbazine, as third-line chemotherapy, was administered for three cycles. In August 2016, the patient was referred to the emergency room for stroke and she died. No necropsy was made and pulmonary embolism was assumed as the causal event.

### Case 3

The case is a 63-year-old woman with a history of hormone-sensitive bilateral breast cancer (stage II) treated with a bilateral quadrantectomy and axillary lymph node dissection. The patient reported a family history of cancer, a brother and sister with gastric cancer, and a nephew with breast neoplasm.

The patient received a combination of adjuvant radiotherapy (50 Gy by photons in 25 fractions + 10 Gy by two 6-MeV tangential electrons beams in 5 fractions) and chemotherapy (epirubicin plus paclitaxel for four cycles and cyclophosphamide plus methotrexate plus fluorouracil for four cycles), hormone therapy with tamoxifen for 1 year, and anastrozole for 5 years to avoid endometrial hyperplasia.

After 11 years of follow-up, clinical examination of breast documented a mass in the residual of excised left breast and the patient was then subjected to left radical mastectomy. Histologically, it was referred to as poorly differentiated (G3) metaplastic carcinoma of the breast with mesenchymal differentiation (MCMD), score 8 according to Elston and Ellis criteria. Areas of high-grade chondrosarcoma, which constitute 30% of the neoplasm, were present. Immunohistochemical characterization revealed positivity for Vimentin and S100, whereas tissue sections were negative for cytokeratin AE1/AE3 and 34beta E12, and p63. Tissue specimens were also estrogen receptor (ER) and progesterone receptor (PR) negative, and slightly positive for HER2. Ki67^+^ cells were 20%. No vascular and neural invasion were observed. TNM staging was rpT2pNx. After surgery, the patient entered a clinical and radiological follow-up program, as she refused adjuvant therapy. Nine months later, a follow-up chest x-ray showed multiple secondary lung lesions unsuitable for surgical excision. After a multispecialty evaluation, based on the absence of symptoms and the palliative intent of treatment, the patient underwent 3 days of ifosfamide continuous infusion; cycles were repeated every 3 weeks. After six cycles, the patient had partial response and she was asymptomatic during the subsequent follow-up period. Thirteen months later, a CT scan showed lung disease progression that required a systemic therapy consisting of 1,000 mg/mq gemcitabine on days 1 and 8, every 3 weeks. The patient did not improve after four cycles of treatment (June 2017). Since then, the patient chose a 1-month rest period from chemotherapy, until a further progression of lung lesions was documented. A third-line chemotherapy regimen, based on continuous infusion of high-dose ifosfamide, was administered for seven consecutive days over 14 days for eight cycles. In July 2018, pulmonary disease further progressed and, after 2 months, the patient died due to respiratory failure.

### Case 4

A 75-year-old woman underwent right quadrantectomy surgery for a pT1cN0, estrogen receptor positive breast cancer. The patient was treated with CMF (cyclophosphamide plus methotrexate plus 5-fluorouracil) in an adjuvant chemotherapy setting and RT of the right breast (50 Gy by photons in 25 fractions + 10 Gy by single direct 9-MeV electron field in 5 fractions), followed by 5 years of anastrozole therapy. Two years later, during a follow-up visit, a left parascapular mass was noticed. Biopsy and radical excision showed moderately differentiated leiomyosarcoma (G2) showing giant cells with morphologically recognizable smooth muscle differentiation, histological grade 6 according to the French Federation of Cancer Centers Sarcoma Group, and pT2a according to TNM staging (7th ed.). Histologically, it was described as a malignant mesenchymal neoplasm composed of spindle cells with a marked cyto-nuclear atypia and eosinophilic poorly defined cytoplasm, organized in parallel bundles. The immunophenotypic profile was as follows: positive for vimentin, SMA, EMA (epithelial membrane antigen), and actin (clone HHF-35), and negative for CK-pan, Melan A, desmin, CD34, and S-100. The patient underwent post-surgery radiotherapy with 200 cGy for 30 fractions. After 14 months of follow-up, a local relapse in the left humerus-scapular region was observed and excised. Histological evaluation defined a high-grade pleomorphic sarcoma with skin ulcerative lesions, infiltrating subcutaneous tissue and showing vascular embolization. Due to the patient’s poor general condition and comorbidities, she was not suitable for further systemic chemotherapy, and, after a period of palliative care, she died.

### Case 5

The patient is a 61-year-old woman with a diagnosis of left breast infiltrating ductal carcinoma, pT1cN0, grade G2, ER 98%, PGR 20%, HER2+, who underwent quadrantectomy and axillary lymph node dissection followed by adjuvant chemotherapy, radiotherapy (50 Gy in 25 fractions + 10 Gy in 5 fractions by photons), and letrozole administration for 5 years. After 6 years of follow-up, the patient was diagnosed with a left breast locally advanced angiosarcoma, for which she received neo-adjuvant chemotherapy. One year later, the patient underwent a left mastectomy. After one month, a new mass was noticed. Nine months onward, the patient had right breast mammography and bilateral ultrasound examination, which showed a new lesion on the right breast along with an ulceration on the left thoracic wall. The patient met with our plastic surgery team and she was then subjected to surgical excision and electrochemotherapy for both lesions. Histological examination documented a high-grade angiosarcoma (G3), positive for Factor VIII and CD31, with extensive areas of necrosis and ulceration. During the last follow-up record, 3 months after surgery, she showed local condition improvement but soon after she died.

### Case 6

The patient is a 53-year-old woman with a left breast triple-negative infiltrating ductal carcinoma, pT2N1 (N+8/24), G3, surgically excised through left radical mastectomy and axillary lymph node dissection. Following the decision of the Multispecialty Tumor Board, adjuvant antracycline–paclitaxel combination regimen was administered. After 12 years, a local relapse (grade 3 invasive adenocarcinoma) infiltrating dermis and muscle tissue and extending to the thoracic wall was diagnosed. The pathologist described a triple-negative breast cancer with Ki67 index at 50%. The patient underwent surgical excision of pectoral muscle and further chemotherapy treatment with CMF was administered. Seven months onward, radicalization surgery was performed. The patient was then subjected to chemotherapy with epirubicin and paclitaxel, and local radiotherapy plus CWB (chest wall boost) (50 Gy in 25 fractions + 10 Gy in 5 fractions by photons).

The patient had regular clinical and radiological follow-up for 14 years until a left axillar mass and enlarged lymph nodes were detected. Biopsies of the left chest wall showed neoplasm from globose cells with highly pleomorphic nuclei immersed in large necrosis areas. The immunophenotypic profile was found to be positive for CD10, desmin, muscle actin HHF-35, and CD68 (occasionally), and negative for Myo D1, SMA, S-100, CK-pan, CD31, CD34, and Factor VIII; Ki67 proliferation index was 50%. On these bases, it was referred to as a high-grade phyllodes tumor or sarcoma with myofibroblastic/pleomorphic differentiation. Disease evaluation with magnetic resonance imaging (MRI) and CT showed an extensive mass with lymph node metastases. The patient received chemotherapy based on epirubicin and ifosfamide. After two cycles, the patient’s conditions deteriorated with massive pleural effusion and chest invasion, which led to the patient’s death.

### Case 7

The case concerns a 63-year-old woman who, in 2011, underwent left breast quadrantectomy and axillary lymph node dissection for infiltrating ductal breast cancer [pT1cN1(1/18), G2, ER: 90%, PGR: 60%, Ki67 index at 15%, and HER2 negative]. Thereafter, the patient received chemotherapy with six cycles of FEC regimen (5-fluorouracil, epidoxorubicin, and cyclophosphamide), radiotherapy (50 Gy in 25 fractions + 10 Gy in 5 fractions by photons), and letrozole for 5 years. During the follow-up, 9 years later, there was evidence of an ulcerated and bleeding left breast lump, 7 cm in diameter, adherent to the chest wall, and a suspect of bilateral secondary pulmonary lesions through total body CT. A biopsy of the lesion documented a morphological picture showing fibrotic tissue and atypical epithelioid cell aggregates that sometimes optically border empty spaces. The absence of Pan-cytokeratin and positivity for vascular markers was reported. Ki67 was positive in 60% of neoplastic cells. The overall picture was traceable to angiosarcoma. The patient received a single radiofrequency thermoablation session on the breast lesion, resulting in suspension of bleeding, and a first-line chemotherapy for radio-induced angiosarcoma based on three cycles of gemcitabine and docetaxel but without benefit. In July 2021, after internal collegial discussion and sharing the case with a Cancer Center specialized in sarcomas, the patient received one electrochemotherapy session and then a second-line chemotherapy based on weekly doxorubicin administration. A new disease evaluation was made after nine chemotherapy cycles; CT images showed stable pulmonary nodes and no new mass onset. The patient was subjected to another session of electrochemotherapy after 6 months. Biopsy showed chronic and acute inflammation with ascending characters and giant cells from foreign body, marked fibrosis, and epidermal atrophy but no evidence of neoplasm. The patient received 15 cycles of chemotherapy. During the last follow up, in March 2022, she has shown stable disease.

## Discussion

Although radiotherapy represents one of the cornerstones in cancer treatment, it has been assessed that RIS could be a complication. Since the interval between the RT and RIS occurrence is long, it is a key point to perform a strict and continuous follow-up to make an early and accurate diagnosis in order to guarantee an adequate treatment. Overall, RIS represented less than 4% of all sarcoma patients, and arose in 0.19% and 0.10% of RT-treated breast and head and neck cancers, respectively. These results are in line with previous reports (5, 12, 17–20). Our cohort of patients showed clinicopathological features similar to those in existing literature (13, 14). In our study, female patients with RIS were about 85% (6/7), according to the high prevalence of primary breast cancer in women (21). In previous studies, a median age of primary tumor diagnosis ranging from 41 to 46 years, a median latency period to RIS from 8 to 14 years, and a median age at RIS presentation ranging from 52 to 59 years have been reported (21). In slight contrast, we found that our patients were older at primary cancer diagnosis (57.3 years) and that they were characterized by a shorter RIS latency period (7.6 years), which also delayed the median age at RIS diagnosis (64.8 years) (14). This shorter latency time might be in part associated with concurrent chemotherapy administered to treat primary tumor, as previously described by Zhang et al. (22). However, the median survival time, 36 months, was found to be quite comparable to that from other reports (14, 23).

Despite their low incidence, RIS is characterized by high aggressiveness from both local and systemic points of view, which results in high mortality rates. Recent reports highlighted the non-inferiority of the hypofractionated radiation regimen as compared with the conventional one (24, 25). Notably, although long-term real-life data on the RIS risk associated with hypofractionated irradiation are lacking, some reports highlight the possible occurrence of secondary cancers in the irradiated field (26, 27). R0 resection is widely considered the only curative chance for these patients (28), although all RIS patients in our case series had tumor relapse after surgical resection. Moreover, our patients received scarce benefits from multiple lines of chemotherapy. However, the poor prognosis of RIS patients did not discourage the employment of radiotherapy, an indispensable therapeutic approach for cancer treatment, since its benefits undoubtedly outweigh the risks.

## Conclusions

RIS is a possible complication of long-survivor cancer patients; thus, much attention has to be paid to early diagnose these cancers to employ optimal lifesaving therapies. Adherence to a strict follow-up regimen after the radiation treatment to assess and mitigate the risk of post-radiation tumor onset is recommended. After the planned follow-up period, considering the long-term risk to develop a RIS, it is also necessary to apply a specific survivorship care plan. Our center is working to organize a multispecialty survivorship program that will include hospital physicians, general practitioners, and outsource experts specialized in supportive discipline, including nutritional support.

## Data availability statement

The raw data supporting the conclusions of this article will be made available by the authors, without undue reservation.

## Ethics statement

Ethical review and approval was not required for the study on human participants in accordance with the local legislation and institutional requirements. The patients/participants provided their written informed consent to participate in this study.

## Author contributions

SR and AMB designed the work. LCO, SL, AB, APS collected data. SL, FA, and SR analysed data. GM and AMB interpreted data. LCO, SL, and SR drafted the work. AS, GF, and AMB substantially revised the work. All authors contributed to the article and approved the submitted version.

## Funding

This study has been funded by Ministero della Salute, Ricerca Corrente 2022.

## Conflict of interest

The authors declare that the research was conducted in the absence of any commercial or financial relationships that could be construed as a potential conflict of interest.

## Publisher’s note

All claims expressed in this article are solely those of the authors and do not necessarily represent those of their affiliated organizations, or those of the publisher, the editors and the reviewers. Any product that may be evaluated in this article, or claim that may be made by its manufacturer, is not guaranteed or endorsed by the publisher.

## References

[1] Mirjolet C Merlin JL Truc G Noël G Thariat J Domont J . RILA blood biomarker as a predictor of radiation-induced sarcoma in a matched cohort study. EBioMedicine (2019) 41:420–6. doi: 10.1016/j.ebiom.2019.02.031 PMC6442988 30827931

[2] Baskar R Lee KA Yeo R Yeoh K-W . Cancer and radiation therapy: Current advances and future directions. Int J Med Sci (2012) 9:193–9. doi: 10.7150/ijms.3635 PMC3298009 22408567

[3] Abshire D Lang MK . The evolution of radiation therapy in treating cancer. Semin Oncol Nurs (2018) 34:151–7. doi: 10.1016/j.soncn.2018.03.006 29606538

[4] Joo MW Kang YK Ogura K Iwata S Kim JH Jeong WJ . Post-radiation sarcoma: A study by the Eastern Asian musculoskeletal oncology group. PLoS One (2018) 13:e0204927. doi: 10.1371/journal.pone.0204927 30332455 PMC6192585

[5] Pradniwat K Ong KW Sittampalam K Bay BH Tan PH . Sarcoma of the breast and chest wall after radiation treatment for bilateral breast carcinoma. J Clin Pathol (2015) 68:491–5. doi: 10.1136/jclinpath-2015-202963 25788453

[6] Tubiana M . Can we reduce the incidence of second primary malignancies occurring after radiotherapy? a critical review. Radiother Oncol (2009) 91:4–15. doi: 10.1016/j.radonc.2008.12.016 19201045

[7] O’Regan K Hall M Jagannathan J Giardino A Kelly PJ Butrynski J . Imaging of radiation-associated sarcoma. AJR Am J Roentgenol (2011) 197(1):W30–6. doi: 10.2214/AJR.10.5558 21700992

[8] Phillips TL Sheline GE . Bone sarcomas following radiation therapy. Radiology (1963) 81:992–6. doi: 10.1148/81.6.992 14101725

[9] Pendlebury SC Bilous M Langlands AO . Sarcomas followig radiation therapy for breast cancer: A report of three cases and a review of the literature. Int J Radiat OncologyBiologyPhysics (1995) 31:405–10. doi: 10.1016/0360-3016(95)93157-3 7836096

[10] Cahan WG Woodard HQ . Sarcoma arising in irradiated bone; report of 11 cases. Cancer (1948) 1:3–29. doi: 10.1002/(sici)1097-0142(19980101)82:1<8::aid-cncr3>3.0.co;2-w 18867438

[11] Wu LC Kleinerman RA Curtis RE Savage SA Berrington de González A . Patterns of bone sarcomas as a second malignancy in relation to radiotherapy in adulthood and histologic type. Cancer Epidemiol Biomarkers Prev (2012) 21:1993–9. doi: 10.1158/1055-9965.EPI-12-0810 PMC3493823 22964827

[12] Snow A Ring A Struycken L Mack W Koç M Lang JE . Incidence of radiation induced sarcoma attributable to radiotherapy in adults: A retrospective cohort study in the SEER cancer registries across 17 primary tumor sites. Cancer Epidemiol (2021) 70:101857. doi: 10.1016/j.canep.2020.101857 33249363 PMC7856279

[13] Sheth GR Cranmer LD Smith BD Grasso-Lebeau L Lang JE . Radiation-induced sarcoma of the breast: a systematic review. Oncologist (2012) 17:405–18. doi: 10.1634/theoncologist.2011-0282 PMC3316927 22334455

[14] Lou J Jiang L Dai X Wang H Yang J Guo L . Radiation-induced sarcoma of the head and neck following radiotherapy for nasopharyngeal carcinoma: A single institutional experience and literature review. Front Oncol (2020) 10:526360. doi: 10.3389/fonc.2020.526360 33552942 PMC7858657

[15] Hothorn T Lausen B . On the exact distribution of maximally selected rank statistics. Comput Stat Data Anal (2003) 43:121–37. doi: 10.1016/S0167-9473(02)00225-6

[16] Therneau TM . A package for survival analysis in r (2022). Available at: https://CRAN.R-project.org/package=survival (Accessed May 31, 2022).

[17] Bjerkehagen B Smeland S Walberg L Skjeldal S Hall KS Nesland JM . Radiation-induced sarcoma: 25-year experience from the Norwegian radium hospital. Acta Oncol (2008) 47:1475–82. doi: 10.1080/02841860802047387 18607853

[18] Taghian A de Vathaire F Terrier P Le M Auquier A Mouriesse H . Long-term risk of sarcoma following radiation treatment for breast cancer. Int J Radiat Oncol Biol Phys (1991) 21:361–7. doi: 10.1016/0360-3016(91)90783-z 1648044

[19] Kirova YM Feuilhade F Calitchi E Otmezguine Y Le Bourgeois JP . Radiation-induced sarcomas following radiotherapy for breast cancer: Six case reports and a review of the literature. Breast (1998) 7:277–82. doi: 10.1016/S0960-9776(98)90096-0

[20] Garcia M Hernandez DL Mendoza S Buelvas N Alvarez A Esguerra J . Tumors associated with radiotherapy: A case series. J Med Case Rep (2020) 14:179. doi: 10.1186/s13256-020-02482-x 33019945 PMC7537103

[21] Callesen LB Safwat A Rose HK Sørensen FB Baad-Hansen T Aggerholm-Pedersen N . Radiation-induced sarcoma: A retrospective population-based study over 34 years in a single institution. Clin Oncol (R Coll Radiol) (2021) 33:e232–8. doi: 10.1016/j.clon.2020.12.009 33386215

[22] Zhang AY Judson I Benson C Wunder JS Ray-Coquard I Grimer RJ . Chemotherapy with radiotherapy influences time-to-development of radiation-induced sarcomas: A multicenter study. Br J Cancer (2017) 117:326–31. doi: 10.1038/bjc.2017.198 PMC5537501 28654633

[23] Cha C Antonescu CR Quan ML Maru S Brennan MF . Long-term results with resection of radiation-induced soft tissue sarcomas. Ann Surg (2004) 239:903–9. doi: 10.1097/01.sla.0000128686.51815.8b PMC1356299 15166970

[24] Lertbutsayanukul C Pitak M Nantavithya C . Long-term oncological outcomes of hypofractionated versus conventional fractionated whole breast irradiation with simultaneous integrated boost in early-stage breast cancer. Radiat Oncol J (2022) 40:141–50. doi: 10.3857/roj.2021.00927 PMC9262705 35796117

[25] Piras A Boldrini L Menna S Venuti V Pernice G Franzese C . Hypofractionated radiotherapy in head and neck cancer elderly patients: A feasibility and safety systematic review for the clinician. Front Oncol (2021) 11:761393. doi: 10.3389/fonc.2021.761393 34868976 PMC8633531

[26] Cook MR Martinez MP Fenger JM Desai NC . Radiation-induced sarcoma in a cat following hypofractionated, palliative intent radiation therapy for large-cell lymphoma. JFMS Open Rep (2019) 5(2):2055116919889159. doi: 10.1177/2055116919889159 31819802 PMC6882035

[27] Zwahlen DR Bischoff LI Gruber G Sumila M Schneider U . Estimation of second cancer risk after radiotherapy for rectal cancer: comparison of 3D conformal radiotherapy and volumetric modulated arc therapy using different high dose fractionation schemes. Radiat Oncol (2016) 11:149. doi: 10.1186/s13014-016-0723-6 27832799 PMC5103599

[28] Giannini L Incandela F Fiore M Gronchi A Stacchiotti S Sangalli C . Radiation-induced sarcoma of the head and neck: A review of the literature. Front Oncol (2018) 8:449. doi: 10.3389/fonc.2018.00449 30386739 PMC6199463

